# Down-regulation of miR-206 is associated with Hirschsprung disease and suppresses cell migration and proliferation in cell models

**DOI:** 10.1038/srep09302

**Published:** 2015-03-20

**Authors:** Ankur Sharan, Hairong Zhu, Hua Xie, Hongxing Li, Junwei Tang, Weibing Tang, Hongwei Zhang, Yankai Xia

**Affiliations:** 1 State Key Laboratory of Reproductive Medicine, Institute of Toxicology, School of Public Health, Nanjing Medical University, Nanjing 211166, China; 2 Key Laboratory of Modern Toxicology (Nanjing Medical University), Ministry of Education, China; 3 Department of Pediatric Surgery, Nanjing Children's Hospital Affiliated to Nanjing Medical University, Nanjing 210008, China; 4 Department of Pediatric Surgery, Xuzhou Children's Hospital Affiliated to Xuzhou Medical University, Xuzhou 221002, China

## Abstract

Hirschsprung disease (HSCR) is a well-known congenital digestive disease that originates due to the developmental disorder of neural crest cells. MiR-206 is kown to have a relationship with digestive malfunctions. Therefore, we investigated whether or not miR-206 was involved in the pathogenesis of HSCR. qRT-PCR and Western blot assays were used to detect the expression levels of miRNA and mRNAs, and proteins in case and control tissue samples and two cell lines (293T and SH-SY5Y). The functions of miR-206 *in vitro* were measured by transwell assay, CCK8 assay and flow cytometry. Finally, we conducted dual-luciferase reporter assay to verify the connections between miR-206 and the target mRNA *SDPR*. Down-regulation of miR-206 was found in HSCR case tissue samples compared with controls, which was validated to be connected with the increased level of mRNA and protein of *SDPR* by qRT-PCR and dual-luciferase reporter assay. Moreover, miR-206 suppressed the cell migration and proliferation and silencing of *SDPR* could rescue the extent of the suppressing effects by miR-206 inhibitor. The findings suggest that miR-206 may play a significant role in the pathogenesis of HSCR, as well as inhibiting the cell migration and proliferation by targeting *SDPR* in disease models.

Hirschsprung disease (HSCR) is a common congenital digestive malformation which is characterized by the absence of the ganglion cells in the sub-mucosal and mesenteric plexuses. The incidence of HSCR is two in 10000 live births worldwide with significant differences in various ethnic groups (1.5, 2.1 and 2.8 per 10000 live births in Caucasians, African-Americans and Asians, respectively)^1^. Still, the mechanism of the pathogenesis of HSCR remains unclear except that during the 5_th_ to 12_th_ week in fetal period, the enteric neural crest cells, strongly associated with the bowel functions, fails to migrate to the hindgut^2^. HSCR is proven to have a complex genetic etiology involving several genes, including *RET, EDNRB, SOX10* and *PHOX2B*^3^,^4^,^5^,^6^. However, there are few reports on the roles of non-coding genes, such as miRNA, in the pathogenesis of HSCR.

MicroRNAs (miRNAs) are endogenous 20 ~ 24 nt RNAs that play significant role in regulating gene expression post-transcriptionally in animals and plants by binding to the 3'UTR of the mRNA of the target genes^7^,^8^. MiRNAs are reported to have strong association with diverse diseases, including malignancies, such as gastric cancer, breast cancer, colon cancer and lung cancer, by affecting the cell migration, metastasis, proliferation and apoptosis^9^,^10^,^11^,^12^. Recent studies have demonstrated that miR-206 is responsible for various cancers due to its impact on the cell biological processes, such as cell proliferation, cell differentiation and apoptosis^13^,^14^,^15^. However, until now, there are no reports on the involvement of miR-206 in the early pathogenesis of HSCR.

In this study, we conducted experiments to unravel how miR-206 interacts with its target gene that contributes to the pathogenesis of HSCR in disease models.

## Methods

### Ethics Statement and subject tissue samples

The study was approved by the Institutional Ethics Committee of Nanjing Medical University. All of the experiments in the research were in compliance with the government policies and defined protocols which are accepted in current practices. In total, 80 HSCR case samples were enrolled into the research, which were earlier diagnosed by barium enema and anorectal manometry evaluation after surgery between 2009 and 2013 (NJMU Birth Cohort). Also, the entire group of control was 80 matched subjects that were confirmed HSCR-free. Written informed consent was obtained from patients' guardians after full explanation of the experiment. All tissue samples were stored at −80°C immediately after surgery.

### RNA extraction and quantitative real-time PCR (RT-PCR)

Total RNAs, including miRNAs, were extracted from 80 matched controls, HSCR-stenosed segments (HSCR-S) and 80 HSCR-dilated segments (HSCR-D) colon tissue samples and two cell lines by the method of Trizol reagent (Life Technologies, CA, US). TaqMan® MicroRNA Assays (Applied Biosystems, CA, US) was applied for the detection of expression level of miR-206 in tissue samples with the normal endogenous control. Meanwhile, the mRNA of *SDPR* was measured by ABI 7900HT with SYBR (Takara, Tokyo, Japan) along with the GAPDH as the internal control. Details of the probes and primers are given in Supplementary Table 1.

### Protein extraction and Western blot

The tissue samples and cells were lysed by RIPA buffer (Beyotime, Nantong, China). The incubation of the primary antibody (cat. # AP9935c, Abgent, SanDiego, US) against *SDPR* proteins with Polyvinylidene Fluoride (PVDF) membranes was performed at 4°C overnight. After rinsing, the secondary antibody (Beyotime, Nantong, China) was incubated with the PVDF membranes for 1 hour at room temperature. During the whole process, GAPDH was regarded as the normal control. Image J software was applied for the detection and quantification of the protein level in Western Blot.

### Cell culture and transfection

In this study, due to the limitation of ENS cell model of HSCR, we searched many papers and found two appropriate cell lines, namely, human 293T and SH-SY5Y cells, which were acquired from American Type Culture Collection (ATCC, Manassas VA, US) were employed for the experiments *in vitro*. Both cell lines were cultured in DMEM medium (Hyclone, UT, US), which contained 10% heat-inactivated fetal bovine serum (FBS), penicillin (100 U/ml) and streptomycin (100 ug/ml) under the condition of 37°C and 5% CO_2_. The miRNA precursor molecules and *SDPR* siRNA used in transfection were synthesized and purchased from GenePharma (Shanghai, China). Lipofectamine 2000 Reagent (Invitrogen, CA, US) was used as the vector for transfection reagents during the procedure according to the manufacturers' protocols.

### Cell transwell and proliferation assay

Cell transwell assay, which was designed to evaluate the capacity of cell migration, was mainly performed with the application of the Transwell migration chambers (8 μm pore size, Millipore Corporation, Billerica, MA). Firstly, two cell lines were cultured in six-well plates and transfected with miR-206 inhibitor/mimics or *SDPR* siRNA. After 48 h of transfection, cells were harvested with serum-free medium as single-cell suspension and 100 μl of cell suspension was seeded in the upper chamber (1 × 10^6^ cells/ml), along with the lower chamber filled with the 600 μl DMEM medium with 10% FBS. After incubation from 24 h to 48 h, the cells were stained with crystal violet staining solution (Beyotime, Nantong, China) after cell fixation in 95% methyl aldehyde. The images of migrated cells were captured and counted by Image-pro Plus 6.0 with the amount of normal control cells standardized to 1. Accompanied with the transwell assay, CCK8 assay was conducted to confirm the cell proliferation after 24 h transfection. Transfected cells were planted into 96-well plates and add CCK8 reagent into each well for 1 h incubation at 37°C, which was evaluated by 450 nm absorption measured by TECAN infinite M200 multimodemicroplate reader (Tecan, Mechelen, Belgium). Experiments of cell transwell and proliferation were performed in triplicate independently.

### Cell cycle and apoptosis assay

To investigate whether miR-206 had any impact on cell cycle and apoptosis, cells transfected with miR-206 inhibitor were harvested and detected by BD Biasciences FACS Calibur Flow Cytometry (BD Biosciences, NJ, US). For detection of apoptosis, after collection, cells were stained with Annexin V-FITC/Propidium Iodide Kit (KeyGen Biotech, Nanjing, China)and data analyzed with FlowJo V7 software (Tree Star, Oregon, US). Experiments of cell cycle and apoptosis were also performed in triplicate independently.

### Dual-luciferase reporter assay

Dual-luciferase reporter assay was used to validate whether or not miR-206 regulated *SDPR* by binding to the 3'UTR region of *SDPR* mRNA. Thus, the sequence of 3'UTR region of *SDPR* predicted to have interaction with miR-206 was inserted into the KpnI and SacI sites of pGL3 promoter vector (Genscript, Nanjing, China). These constructs were named pGL3-*SDPR* and pGL3-*SDPR*-mut, respectively. According to the manufacturers' protocols, after transfection with negative control, miR-206 mimics, pGL3-*SDPR* and pGL3-*SDPR*-mut, cells were collected to measure fire fly and renilla luciferase activities by Dual Luciferase Assay (Promega, Madison, WI). Experiments of dual-luciferase reporter assay were also performed in triplicate independently.

### Statistical analysis

Statistical analyses were performed by using Stata 9.0 statistical software package (Stata Corp. Texas, US) and presented by Graphpad software (GraphPad Software, Inc., CA, US). Data of the relative expression level of miR-206 and *SDPR* in human tissue samples were presented as a box plot of the median and range of log-transformed expression level accessed by Wilcoxon rank-sum test. The data for the experiments *in vitro* that were repeated three times, were plotted as mean ± SEM via double-sided Student's t-test. Results were considered to have statistically significant differences if *p* < 0.05.

## Results

### Study population

In total, 160 colon tissue specimens were recruited from Department of Pediatric Surgery, Nanjing Children's Hospital affiliated Nanjing Medical University, including 80 HSCR-confirmed cases and 80 matched controls. The clinical characteristics of the study subjects are shown in Table 1,ranging over age, sex and disease classification. As displayed in Table 1, there were no statistically significant differences in terms of age and sex between HSCR cases and controls. Moreover, according to the length of aganglionosis in colon, HSCR was divided into two main types, short-segment HSCR (S-HSCR) and long-segment HSCR (L-HSCR).

### Down-regulation of miR-206 in HSCR

**Figure 1:**
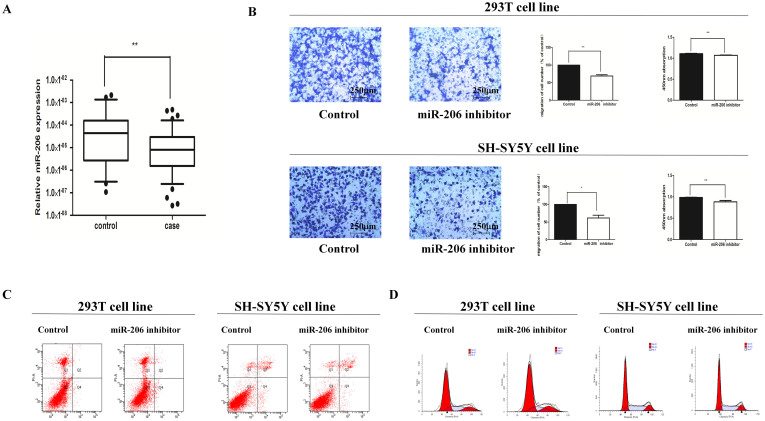
MiR-206 was down-regulated and its cell biological change after treating cell lines with miR-206 inhibitor. (A): The relative expression levels of miR-206 in human HSCR tissues (n = 80) and control tissues (n = 80) were evaluated by qRT-PCR. Data were presented as box plot of the median and range of log-transformed relative expression levels. The top and bottom of the box represent the seventy-fifth and twenty-fifth percentile. The whiskers indicate the 10th and 90th points. * Significantly different compared with that of control (P < 0.05). (B): Transwell assay was performed as described in Materials and Methods. The representative images of invasive cells at the bottom of the membrane stained with crystal violet were visualized as shown (left). The quantifications of cell migration were presented as percentage migrated cell numbers (right). * indicates significant difference compared with control group (P < 0.05). Absorbance at 450 nm was presented with Mean ± SE. * indicates significant difference compared with control group P < 0.05. (C–D): Flow cytometry assay was performed to evaluate cell apoptosis and cell cycle.

Figure 1A shows that the relative expression level of miR-206 in HSCR cases was significantly lower as compared with matched controls. This implies that miR-206 might have connections with the pathogenesis of HSCR. Another experiment was conducted to examine the miR-206 expression level in 80 matched controls, HSCR-stenosed segments (HSCR-S) and 80 HSCR-dilated segments (HSCR-D). The results showed that the expression level of HSCR-D and HSCR-S were both much lower than controls (Supplement Figure C).

### MiR-206 inhibitor suppressed cell migration and proliferation without impacting cell cycle and apoptosis

In order to confirm the functional performance of miR-206 in vitro, we examined how miR-206 impacted cell migration, cell proliferation, cell cycle process and apoptosis. To achieve this, 293t and SH-SY5Y cell lines were transfected with miR-206 inhibitor and then subjected to transwell and CCK8 assays. Both the cell lines showed reduction in number of migrating and proliferating cells suggesting that the down-regulation of miR-206 had a suppressive affection on cell migration and proliferation (Figure 1B). Furthermore, flow cytometry analysis was performed to investigate the impact of miR-206 on cell cycle and apoptosis. The results show no statistical difference in the percentage of apoptotic cells between cells transfected with miR-206 inhibitor and the negative control. Likewise, there were no changes in the cell cycle process (Figure 1C,D).

### Bioinformatics prediction of target gene for miR-206

We applied three main databases (DIANA LAB, Targetscan and Pictar) to predict the underlying target genes, which may be regulated by miR-206. Finally, after the prediction and function analysis,the three common target genes, *SDPR*(serum deprivation response), *FN1*(fibronectin 1) and *PAX3*(paired box 3), were selected. *SDPR* and *FN1* are admitted for the confirmed remarkable associations with plasma membrane, which contributes to the dysfunction of caveolae, cell adhesion and migration. *PAX3* is generally accepted to have a key role in the fetal development and pathogenesis of colonic aganglionosis.

### Up-regulation of *SDPR* in HSCR patients

**Figure 2:**
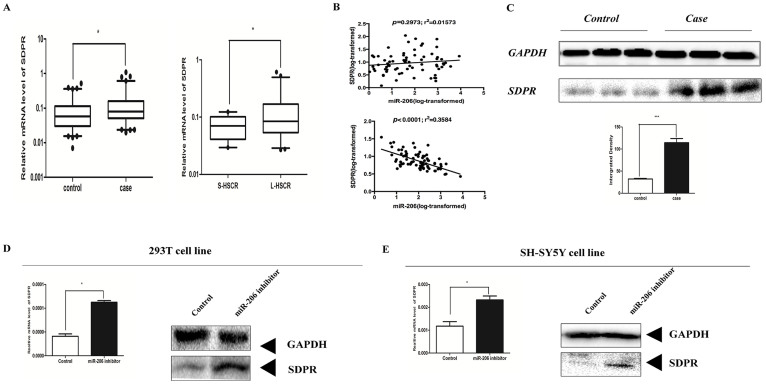
SDPR was up-regulated in HSCR cases and two cell lines. (A): The mRNA expression levels of SDPR in human HSCR case and control tissues and the relative expression level of SDPR in S-HSCR and L-HSCR. (B): Correlation analysis between miR-206 and SDPR in controls and cases, respectively. The upper correlation was performed in controls and it showed poor relationships between miR-206 and SDPR, while the lower correlation analysis indicated that miR-206 is connected with SDPR in HSCR cases. (C): The protein expression levels of PTEN in human HSCR tissues and controls (3 representative samples from both groups are shown) (above). Quantization of Western-blotting was done by Image J software (blow). (D–E): Cells were transfected with 100 nM miR-206 inhibitor for 48 h, qRT-PCR was performed to evaluate the mRNA level of SDPR. SDPR protein expression levels were analyzed by western-blotting.

To determine whether all of three target genes were involved in HSCR, qRT-PCR was used for the examination of the mRNA level in 80 HSCR case and matched control tissue samples. *SDPR* was the only candidate gene that showed significant up-regulation between HSCR cases and matched controls. Moreover, in order to reveal whether expression level of *SDPR* had a relationship with the diseases classification, we checked the expression level of *SDPR* in two main types of HSCR. The results indicate that *SDPR* expressed much more in L-HSCR than S-HSCR (p = 0.0241) (Figure 2A). Immediately, correlation analysis was conducted between miR-206 and *SDPR* in match controls and cases, respectively. The findings demonstrated that compared with the poor correlation in controls, there were evident associations between miR-206 and *SDPR* in HSCR cases (Figure 2B). Simultaneously, via western blot, the protein expression level of *SDPR* was consistent with the mRNA expression level (Figure 2C).

In contrast, FN1 and PAX3 were invariant between HSCR cases and controls (Supplementary Figure A, B). In addition, we evaluated the changes of expression level of *SDPR* in 293T and SH-SY5Y cell lines after transfection with miR-206 inhibitor. After 48 hours, the expression level of *SDPR* mRNA and the protein level were detected by qRT-PCR and western blot, respectively. As expected, *SDPR* expression was remarkably up-regulated at both mRNA and protein levels in 293T and SH-SY5Y cell lines (Figure 2D, E).

### *SDPR* was target gene for miR-206

**Figure 3:**
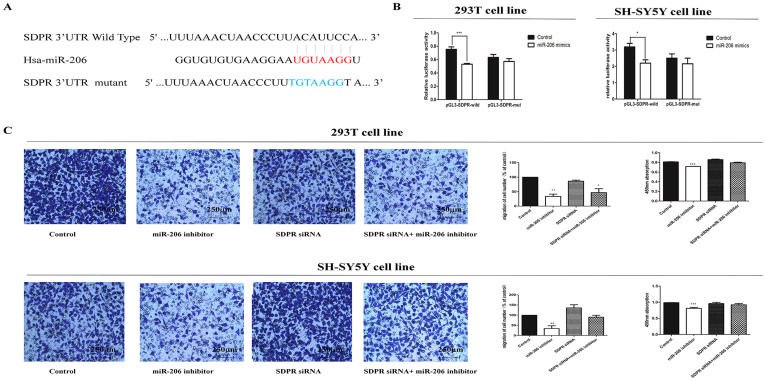
Migration was reversed after co-transfected with miR-206 inhibitor and SDPR siRNA. (A): Sequence alignment of human miR-206 with 3' UTR of SDPR. Bottom: mutations in the 3'-UTR of SDPR in order to create the mutant luciferase reporter construct. (B): Cells were transfected with miR-206 and control, renilla luciferase vector pRL-SV40 and SDPR3'UTR luciferase reporters for 48 h. Both firefly and Renilla luciferase activities are measured in the same sample. Firefly luciferase signals were normalized with Renilla luciferase signals. All tests were performed in triplicate and presented as mean ± SE. (C): The cell biological changes in migration and proliferation were reversed after co-transfected miR-206 inhibitor and SDPR siRNA. Cells were divided into four groups in two cell lines, respectively. The first group was transfected with control, followed by miR-206 inhibitor, SDPR siRNA and the last one that co-transfected with miR-206 and SDPR siRNA. We compared the co-transfected group with others and found migration and proliferation were partly reversed after co-transfected with miR-206 inhibitor and SDPR siRNA when compared with the cells transfected with miR-206 inhibitor, and were much more restored after co-transfected with miR-206 inhibitor and SDPR siRNA. Data were presented as mean ± SEM from three separate experiments performed in triplicates, and were analyzed by double-sided Student's t-test. (* indicates P < 0.05).

To verify the relationship between miR-206 and *SDPR*, two independent methods were applied to validate the miRNA-target gene interaction. Firstly, we constructed the wild and mutant type luciferase plasmids with the binding area of 3'UTR of *SDPR* mRNA, which was referred to as pGL3-*SDPR* and pGL3-*SDPR*-mut, respectively (Figure 3A). Transfection of miR-206 mimics with pGL3-*SDPR* into 293T cell line and SH-SY5Y cell lines significantly inhibited the luciferase activity as compared with the control. Meanwhile, there was no significant alteration in luciferase activity for cell lines transfected with negative control, miR-206 mimics and pGL3-*SDPR*-mut (Figure 3B). The findings demonstrate that miR-206 regulated *SDPR* by combining the 3'UTR region of its mRNA.

### Silencing of *SDPR* partially rescued the cell migration and proliferation with miR-206 inhibitor mediation

We had validated the fact that miR-206 interacted with *SDPR* by binding to its 3'UTR region and the suppressed affections of miR-206 on cell migration and proliferation. In order to further verify whether miR-206 had impact on cell migration and proliferation through *SDPR* directly, we performed a series of rescue experiments. Specific small interfering RNAs (siRNAs) were designed to silence the expression of *SDPR* in two cell lines. The capacity of cell migration transfected with *SDPR* siRNAs was familiar with the normal control. Accompanied with the transfection of miR-206 inhibitor, the silencing of *SDPR* by specific siRNAs could partially draw up the suppression of cell migration, which was still weaker than the normal control. Furthermore, the parallel results from CCK8 assay were displayed after co-transfection with miR-206 inhibitor and *SDPR* siRNAs were performed (Figure 3C). These results clearly indicated that cell migration and proliferation were impaired by up-regulation of *SDPR* due to down-regulation of miR-206.

## Discussion

In our study, three potential target genes of miR-206 were predicted by DIANA LAB, Pictar and Targetscan, while the results showed *SDPR* was the only one that had significant differences between HSCR cases and matched controls. In the following step, we detected the expression level of miR-206 and *SDPR* mRNA in HSCR case and control tissue samples. The results show that miR-206 was down-regulated in HSCR with concomitant up-regulation of *SDPR*. Therefore, dual-luciferase reporter assay was conducted to reveal the underlying relationships between miR-206 and *SDPR*. As expected, the results illustrate that miR-206 had an inverse regulatory relationship with *SDPR* by directly binding to the 3'UTR region of *SDPR* mRNA, which might have caused degradation or structural changes leading to aberrant expression of proteins.

There are many reports suggesting that miR-206 had strong association with regulation of cellular and biological processes. Especially miR-206 was observed to regulate cell movement during zebrafish gastrulation by regulating mitogen-activated protein kinase (MAPK) JNK signaling^16^,^17^. In order to confirm the functional effect of miR-206 on cell biological processes in HSCR, assays such as, cell transwell, cell proliferation, apoptosis and cell cycle were used for validation. The results indicate that the down-regulation of miR-206 suppressed cell migration and proliferation. Moreover, the successful rescue in transwell assay by *SDPR* siRNA implied that miR-206 performed the suppression by up-regulating *SDPR*.

*SDPR* (serum deprivation response) is a key substrate for protein kinase C (PKC) phosphorylation and this interaction determines the compartimentalization of PKC to caveolae^18^. SDPR was further verified to play a key role in inducing membrane curvature and participate in the formation of caveolae^19^. Caveolae is calcium channel related to gut electrophysiological pacing function, which has also been identified to have impacts on cell migration and proliferation^20^,^21^. Recently, it has been demonstrated that the absence of the caveolae on the membrane surface contributed to the formation of fibroblast-like ICC (Interstitial Cajal Progenitors Cells) in the narrow segment of HSCR compared with the normal adult colon, which was shown to have surface caveolae^22^. Meantime, fibroblast-like ICC was observed in Igf1r+/CD34+ ICC in Ws/Ws rat colon^23^. Accordingly, the over-expression of *SDPR* was validated to play a key role in inducing deformation of caveolae and extensive tubulation of the plasma membrane^19^. In our study, the expression level of SDPR in HSCR cases was much higher than the normal matched controls. Thus, we speculate that through negative regulation, down-regulation of miR-206 led to the up-regulation of *SDPR* inducing the deformation of caveolae of ENCCs (enteric neural crest cells) in colon, which would contribute to the pathogenesis of HSCR. Further research needs to be performed to validate the hypothesis, especially the caveolae of ENCCs.

In conclusion, our research reveals that miR-206 plays an important role in the pathogenesis of HSCR and suppresses cell proliferation and migration by regulating *SDPR* in disease models. Our study provides a new approach for understanding the pathogenesis of HSCR and might contribute to a novel approach to the therapy of HSCR in the future.

## Author Contributions

A.S., H.L. and Y.X. designed the experiments; A.S., H.Z. and H.X. performed the experiments; W.T., H.L., J.T. and H.W.Z. contributed essential technical assistance; H.Z. and A.S. wrote the paper.

## Supplementary Material

Supplementary Information Supplementary Information

## Figures and Tables

**Table 1:** Clinical characteristics of the study population

Variable	HSCR (n = 80)	Control (n = 80)	*P*
**Age(months, mean, SE)**	4.25(0.30)	4.77(0.83)	0.57^a^
**Sex**			
**Male**	70	55	0.17^b^
**Female**	10	25	
**Disease Classifcation**			
**S-HSCR**	29		
**L-HSCR**	51		

^a^Student t-test

^b^Two-sided χ^2^ test.

S-HSCR: Short-segment HSCR; L-HSCR: Long-segment HSCR.
