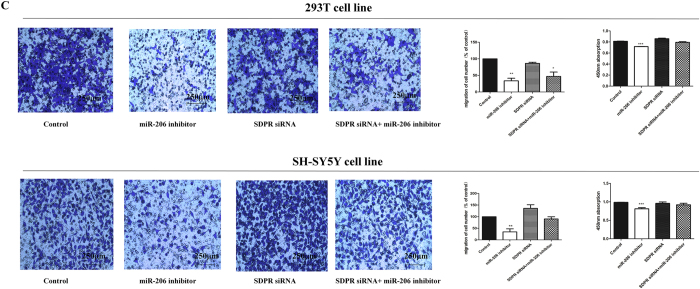# Corrigendum: Down-regulation of miR-206 is associated with Hirschsprung disease and suppresses cell migration and proliferation in cell models

**DOI:** 10.1038/srep17666

**Published:** 2016-01-06

**Authors:** Ankur Sharan, Hairong Zhu, Hua Xie, Hongxing Li, Junwei Tang, Weibing Tang, Hongwei Zhang, Yankai Xia

Scientific Reports 5: Articles number: 9302; 10.1038/srep09302 published online: 03202015; updated: 01062016

This Article contains errors in Figure 3C. The image depicting the 293T cell line co-infected with SDPR siRNA is a duplicate of the Control image of the SH-SY5Y cell line. In addition, the image depicting the 293T cell line co-infected with SDPR siRNA + miR-206 inhibitor is a duplicate of the image depicting the SH-SY5Y cell line co-infected with miR-206-inhibitor. The correct Figure 3C appears below as Figure 1.

## Figures and Tables

**Figure 1:**