# Supplementary material for: Down-regulation of miR-206 is associated with Hirschsprung disease and suppresses cell migration and proliferation in cell models
Source: Sci Rep. 2015 Mar 20;5:9302. doi: 10.1038/srep09302 (PMC4366810; doi:10.1038/srep09302)
Supplement: Supplementary Information [file srep09302-s1.pdf]

# **Down-regulation of miR-206 is associated with Hirschsprung disease and suppresses cell migration and proliferation in cell models**

Ankur Sharan[b,c,#], Hairong Zhu[a,c,#], Hua Xie[a,c], Hongxing Li[a,c], Junwei Tang[a,c], Weibing Tang[a,c], Hongwei Zhang[d], Yankai Xia[a,b]

[a]State Key Laboratory of Reproductive Medicine, Institute of Toxicology, School of Public Health, Nanjing Medical University, Nanjing 211166, China

[b]Key Laboratory of Modern Toxicology (Nanjing Medical University), Ministry of Education, China

[c]Department of Pediatric Surgery, Nanjing Children's Hospital Affiliated to Nanjing Medical University, Nanjing 210008, China.

[d]Department of Pediatric Surgery, Xuzhou Children's Hospital Affiliated to Xuzhou Medical University, Xuzhou 221002, China.

**A**

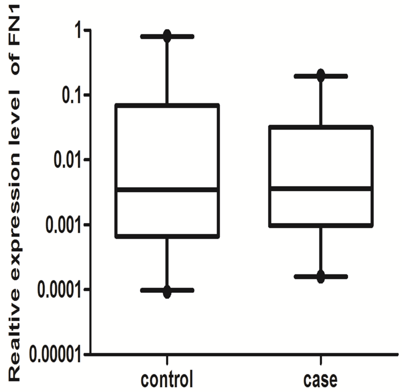
Realtive expression level of FN1
1
0.1
0.01
0.001
0.0001
0.00001
control
case

**B**

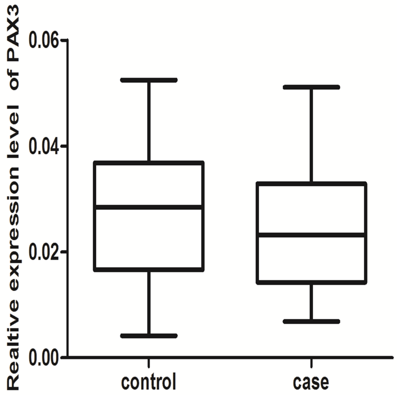
Realtive expression level of PAX3
0.06
0.04
0.02
0.00
control
case

**C**

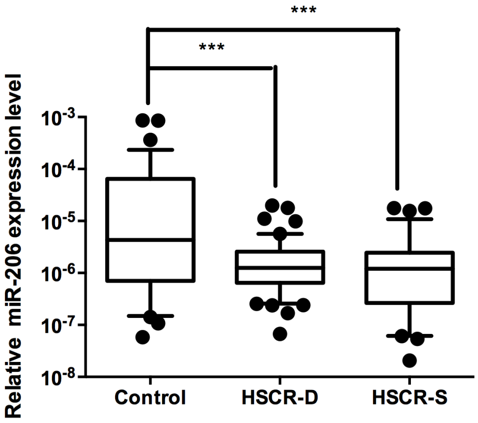
***
***
Relative miR-206 expression level
$10^{-3}$
$10^{-4}$
$10^{-5}$
$10^{-6}$
$10^{-7}$
$10^{-8}$
Control
HSCR-D
HSCR-S

Supplementary figure A-C. No significant differences between HSCR cases and matched controls in the expression level of FN1 and PAX3 mRNA. Extra expression level of miR-206 in controls, HSCR-stenosed segments (HSCR-S) and HSCR-dilated segments (HSCR-D).

**A-B**: The relative expression levels of FN1 and PAX3 in human HSCR tissues (n=80) and control tissues (n=80) were evaluated by qRT-PCR. Data were presented as box plot of the median and range of log-transformed relative expression levels. The top and bottom of the box represent the seventy-fifth and twenty-fifth percentile. The whiskers indicate the 10th and 90th points. * Significantly different compared with that of control ($P<0.05$). **C**: QT-PCR was conducted to investigate the expression level of miR-206 in 80 matched controls, HSCR-stenosed segments (HSCR-S) and 80 HSCR-dilated segments (HSCR-D). The results showed that the expression level of HSCR-D and HSCR-S were both much lower than controls.
